# Trypanosoma brucei Pex13.2 Is an Accessory Peroxin That Functions in the Import of Peroxisome Targeting Sequence Type 2 Proteins and Localizes to Subdomains of the Glycosome

**DOI:** 10.1128/mSphere.00744-19

**Published:** 2020-02-19

**Authors:** Logan P. Crowe, Christina L. Wilkinson, Kathleen R. Nicholson, Meredith T. Morris

**Affiliations:** a Eukaryotic Innovations Center, Department of Genetics and Biochemistry, Clemson University, Clemson, South Carolina, USA; University at Buffalo

**Keywords:** Pex13, *Trypanosoma brucei*, glycosome, kinetoplastids, parasites, peroxisome

## Abstract

Kinetoplastid parasites, including Trypanosoma brucei, Trypanosoma cruzi, and *Leishmania*, harbor unique organelles known as glycosomes, which are evolutionarily related to peroxisomes. Glycosome/peroxisome biogenesis is mediated by proteins called peroxins that facilitate organelle formation, proliferation, and degradation and import of proteins housed therein. Import of matrix proteins occurs via one of two pathways that are dictated by their peroxisome targeting sequence (PTS). In PTS1 import, a C-terminal tripeptide sequence, most commonly SKL, is recognized by the soluble receptor Pex5. In PTS2 import, a less conserved N-terminal sequence is recognized by Pex7. The soluble receptors deliver their cargo to the import channel consisting minimally of Pex13 and Pex14. While much of the import process is conserved, kinetoplastids are the only organisms to have two Pex13s, Pex13.1 and Pex13.2. It is unclear why trypanosomes require two Pex13s when one is sufficient for most eukaryotes. To interrogate the role of Pex13.2, we have employed biochemical approaches to partially resolve the composition of the Pex13/Pex14 import complexes in T. brucei and characterized glycosome morphology and protein import in Pex13.2-deficient parasites. Here, we show that Pex13.2 is an integral glycosome membrane protein that interacts with Pex13.1 and Pex14. The N terminus of Pex13.2 faces the cytoplasmic side of the membrane, where it can facilitate interactions required for protein import. Two-dimensional gel electrophoresis revealed three glycosome membrane complexes containing combinations of Pex13.1, Pex13.2, and Pex14. The silencing of Pex13.2 resulted in parasites with fewer, larger glycosomes and disrupted glycosome protein import, suggesting the protein is involved in glycosome biogenesis as well as protein import. Furthermore, superresolution microscopy demonstrated that Pex13.2 localizes to discrete foci in the glycosome periphery, indicating that the glycosome periphery is not homogenous.

**IMPORTANCE** Trypanosoma brucei causes human African trypanosomiasis and a wasting disease called Nagana in livestock. Current treatments are expensive, toxic, and difficult to administer. Because of this, the search for new drug targets is essential. T. brucei has glycosomes that are essential to parasite survival; however, our ability to target them in drug development is hindered by our lack of understanding about how these organelles are formed and maintained. This work forwards our understanding of how the parasite-specific protein Pex13.2 functions in glycosome protein import and lays the foundation for future studies focused on blocking Pex13.2 function, which would be lethal to bloodstream-form parasites that reside in the mammalian bloodstream.

## Abstract (precis)

Trypanosoma brucei causes human African trypanosomiasis and a wasting disease called Nagana in livestock. Current treatments are expensive, toxic, and difficult to administer. Because of this, the search for new drug targets is essential. T. brucei has glycosomes that are essential to parasite survival; however, our ability to target them in drug development is hindered by our lack of understanding about how these organelles are formed and maintained. This work forwards our understanding of how the parasite-specific protein Pex13.2 functions in glycosome protein import and lays the foundation for future studies focused on blocking Pex13.2 function, which would be lethal to bloodstream-form parasites that reside in the mammalian bloodstream.

## INTRODUCTION

Trypanosoma brucei is a protozoan parasite that causes African trypanosomiasis (HAT) in humans and a wasting disease called Nagana in cattle. The parasite alternates between a mammalian host where it spends much of its time in the bloodstream as a bloodstream-form (BF) parasite and the tsetse fly as a procyclic-form (PF) parasite. Glycosomes are essential, parasite-specific membrane-bound organelles whose composition and function change during development (1) and in response to the environment (2). Small molecules that interfere with the protein interactions that regulate glycosome function are lethal to parasites (3); thus, these organelles and the processes that regulate them are attractive drug targets.

Glycosomes are related to peroxisomes and share many components of the machinery that facilitate protein import into these organelles. Peroxisome and glycosome biogenesis are regulated by proteins called peroxins (Pexs) that govern organelle formation, proliferation, and degradation as well as protein import into the organelle (4–6). Peroxisome and glycosome import involve binding of soluble receptor proteins, either Pex5 or Pex7, to a targeting sequence in the cargo protein (7, 8). Pex5 binds to a C-terminal tripeptide with the consensus sequence SKL called a peroxisome targeting sequence 1 (PTS1), while Pex7 binds to a less conserved N-terminal sequence termed PTS2 (4, 9, 10). The PTS1 and PTS2 receptor-cargo complexes both dock at the peroxisome membrane through interactions with the glycosome membrane proteins Pex13 and Pex14, which make up the import channel (11). After import, the receptors are recycled via a ubiquitination process involving Pex2, -10, and -12 (12).

Kinetoplastids are unique in that they have two Pex13s, which have been designated Pex13.1 and Pex13.2 (13, 14). These proteins share low sequence identity with each other or with Pex13s from higher eukaryotes. In previous studies, Pex13.1 localized to glycosomes. Silencing of the protein in BF and PF parasites yielded parasites with glycosome protein import defects and lowered growth rates (14). Later, iterative database searches resulted in the identification of Pex13.2 (13). Silencing of this second Pex13 via RNA interference (RNAi) in BF parasites resulted in mislocalization of Pex14 and aldolase and a defect in the growth rate. Prior to our work, Pex13.2 RNAi cell lines could not be established in PF parasites (13). Because glycosomes harbor a majority of the proteins involved in glycolysis, which is essential in BF, it is difficult to study Pex13.2 function in that life stage, as many of the expected phenotypes are likely lethal.

Here, we have partially resolved the topology of Pex13.1 and Pex13.2, identified several import protein complexes in PF parasites, and characterized Pex13.2-deficient PF cell lines. Results herein indicate that Pex13.2 interacts with known proteins of the protein import channel that form several high-molecular-weight membrane complexes and is essential for the efficient import of PTS2 sequences.

## RESULTS

### Pex13.2 is an integral glycosomal membrane protein with its N terminus exposed to the cytosol.

**FIG 1:**
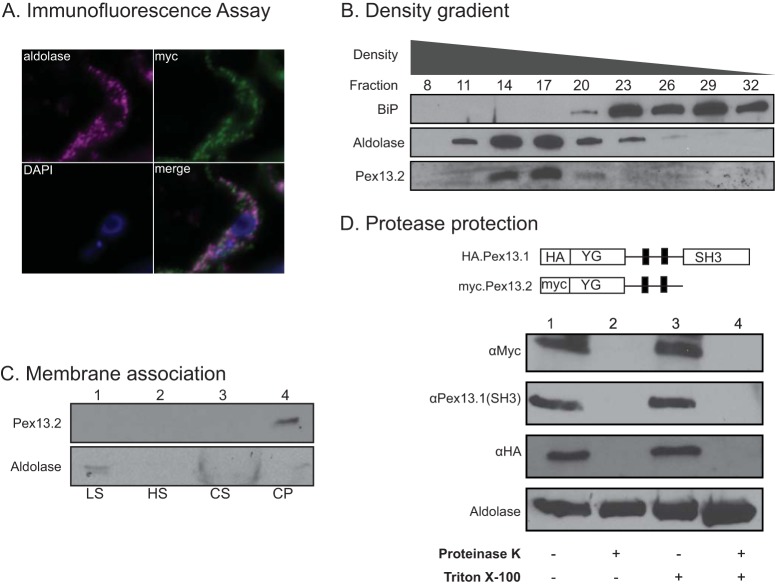
Defining Pex13.2 localization and membrane orientation. (A) IFA was performed on cells expressing epitope-tagged myc.Pex13.2, and images were taken using a Zeiss Axiovert 200M wide-field fluorescence microscope. Aldolase staining is shown in magenta, myc staining shown in green, and DAPI (4′,6-diamidino-2-phenylindole) staining shown in blue. (B) Postnuclear cell lysate was centrifuged through an OptiPrep sucrose gradient, and 1-ml fractions were collected from the bottom of the gradient. Protein was precipitated, separated by SDS-PAGE, and analyzed by Western blot with antibodies against aldolase, BiP, and Pex13.2. (C) Membrane protein extraction of whole-cell membranes. Cells (10^7^) were sequentially extracted with low salt, high salt, and sodium carbonate (LS, HS, and CS, respectively). Proteins were precipitated from supernatants and the final carbonate pellet (CP), separated by SDS-PAGE, transferred to nitrocellulose, and analyzed by Western blotting with antibodies against Pex13.2 and aldolase. (D) Protease protection assay. Cell lysates (lane 1) were treated with proteinase K (lane 2), Triton X-100 (lane 3), or Triton X-100 and protease K (lane 4). Samples were resolved by SDS-PAGE and analyzed by Western blots with anti-myc, anti-Pex13.1 (SH3), and anti-HA antibodies.

To define Pex13.2 localization in PF parasites, we used immunofluorescence assays of cells expressing Pex13.2 fused to a myc epitope tag (myc.Pex13.2). Antibodies against the glycosome protein aldolase and myc both labeled punctate structures consistent with glycosome staining (Fig. 1A). To complement these microscopy studies, we took a biochemical approach to resolve Pex13.2 localization. We isolated organelles via density gradients and analyzed fractions by Western blotting with antibodies made against recombinant Pex13.2, the endoplasmic reticulum (ER) protein BiP, and aldolase. The ER is less dense than glycosomes and equilibrates higher on the gradient. As expected for a glycosome protein, Pex13.2 was detected in fractions 14 to 20 also containing aldolase (Fig. 1B), while BiP was detected in fractions 20 to 32.

Pex13.2 is predicted to have at least 2 transmembrane (TM) domains (13, 15). To confirm that it is an integral membrane protein, we sequentially extracted membrane fractions with low salt, high salt, and sodium carbonate (Fig. 1C). The soluble glycosome matrix protein aldolase was detected in the low-salt supernatants. In contrast, Pex13.2 was detected only in the pellet following sodium carbonate extraction, consistent with being an integral membrane protein.

Most Pex13s in other organisms have both the N and C termini exposed to the cytoplasm (16–18), and previous work indicated that the C-terminal SH3 domain of Pex13.1 also faced the cytosol (14). The topology of Pex13.2 is unknown, and there is no information on whether the N terminus of Pex13.1 faces the cytoplasm or glycosome matrix. We used protease protection assays to assess the orientation of the N termini of both Pex13s. In these experiments, we used cells expressing N-terminally tagged Pex13s (myc.Pex13.2 and HA.Pex13.1) (Fig. 1D). Several attempts at tagging the C termini of either Pex13 were unsuccessful. Treatment of cells with proteinase K (PK) in the absence of Triton X-100 resulted in the loss of myc and hemagglutinin (HA) signal, indicating that the N termini of both proteins were accessible to PK and exposed to the cytosol (Fig. 1D). Pex13.1 antibodies were generated against the SH3 domain of the protein, which was shown to reside on the cytosolic face of the glycosome membrane (14). In our protease protection assays, the SH3 domain of HA.Pex13.1 was degraded by PK in the absence of Triton X-100, indicating that the topology of the Pex13.1 is not altered by addition of the HA epitope tag. As a control, we used aldolase, a matrix protein with a protease-resistant core (19). Full-length aldolase was detected in PK treatments without detergent, indicating that glycosome integrity was not compromised during treatment. After treatment with PK and detergent, we observed a smaller proteolytic product as seen in previous studies documenting the protease-resistant nature of aldolase (19). These results indicate that the N termini of Pex13.1 and Pex13.2 and the C terminus of Pex13.1 are on the cytosolic side of the glycosome membrane.

**FIG 2:**
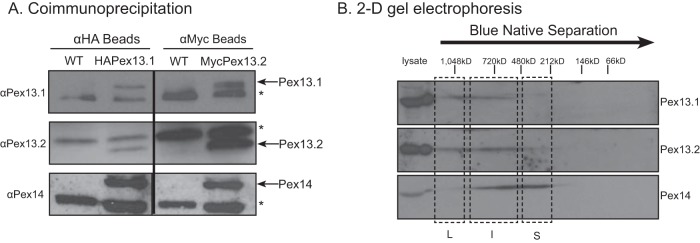
Analysis of complexes formed by Pex13.2. (A) Coimmunoprecipitation experiments were performed by incubating cell lysate of cells coexpressing HA.Pex13.1 and myc.Pex13.2. Lysates were incubated with either anti-HA or anti-myc magnetic beads, and bound proteins were analyzed by SDS-PAGE and Western blotting. (B) Analysis of complexes containing Pex13.1, Pex13.2, and Pex14 by blue native (BN)-PAGE and Western blotting. Wild type (WT) cells were mechanically lysed, and the glycosomal fraction was obtained by differential centrifugation. The glycosome fraction was solubilized in *n*-dodecyl β-maltoside-containing sample buffer, resolved by first-dimension BN-PAGE, and subunits were resolved by second dimension SDS-PAGE and analyzed by Western blotting.

Yeast two-hybrid experiments suggested that Pex13.1 and Pex13.2 might interact (13). We used coimmunoprecipitations (co-IPs) to determine if these proteins bind to each other and with another import complex protein, Pex14, in parasites. Lysates from cells expressing HA.Pex13.1 and myc.Pex13.2 were incubated with anti-HA or anti-myc magnetic beads, and bound proteins were analyzed by Western blotting. Pex13.2 and Pex14 were detected in anti-HA co-IP of cells expressing HA.Pex13.1/myc.Pex13.2 but not in parental PF 2913 cells (Fig. 2A, left). Additionally, Pex13.1 and Pex14 were detected in anti-myc co-IPs of HA.Pex13.1/myc.Pex13.2 cells but not parental 2913 cells (Fig. 2A, right). These data demonstrate that Pex13.1, Pex13.2, and Pex14 interact in parasites.

We next analyzed Pex13.1-, Pex13.2-, and Pex14-containing protein complexes in PF 2913 cells using 2-dimensional gel electrophoresis. Lysates were resolved in the first dimension by blue native gel electrophoresis and in the second dimension by SDS-PAGE. Western blotting revealed the presence of three complexes (Fig. 2B), where the largest complex (L) contained Pex13.1 and Pex13.2, the intermediate complex (I) contained Pex13.1, Pex13.2, and Pex14, and the smaller complex (S) contained primarily Pex14.

### Silencing of Pex13.2 did not slow PF growth but affected glycosome size, number, and density.

**FIG 3:**
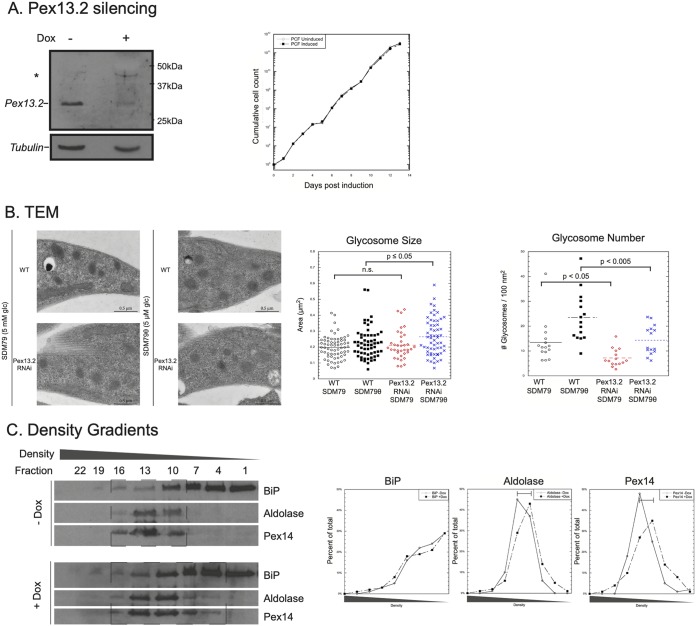
Silencing of Pex13.2 by RNAi results in glycosome morphology changes. (A) Cells harboring the pZJM vector containing a fragment of Pex13.2 were cultured in the absence or presence of doxycycline to induce RNAi expression. Knockdown was confirmed by Western blotting (left). Growth rate was monitored of cells cultured with or without doxycycline (right). (B) Wild-type and RNAi-induced cells were subjected to TEM analysis. (Left) Representative images of WT and Pex13.2 RNAi-induced cells grown in either SDM79 or the low-glucose medium SDM79θ. Glycosomes were measured for size (middle) as well as number of organelles (right). (C) Density gradient centrifugation was performed on both uninduced and induced cells; fractions were taken from the top of the gradients, and protein (2.5 μg) was precipitated and analyzed by SDS-PAGE and Western blotting. Densitometry was used to quantify the protein signal in each fraction relative to the total protein signal (right).

To gain insight into the specific function Pex13.2 plays in glycosome biogenesis, we generated PF Pex13.2-deficient parasites using tetracycline-inducible RNA interference (33). Upon addition of doxycycline (Dox), Pex13.2 expression was reduced 96.1% ± 1.4% (Fig. 3A). Cells were counted daily after induction, and we observed no growth defects in Pex13.2-deficient cells. We next used transmission electron microscopy (TEM) to determine if glycosome number or size was altered upon depletion of Pex13.2 (Fig. 3B). Cells grown in the absence or presence of Dox had 13.5 glycosomes/100 nm^2^ and 7.2 glycosomes/100 nm^2^, respectively. Cells grown without Dox had glycosomes with an average area of 0.20 μm^2^, while those grown with Dox had an average area of 0.21 μm^2^. Under standard culturing conditions in SDM79 medium (5 mM glucose), the disruption of normal glycosome function is often lethal, making phenotypic characterization difficult. However, when cells are grown in media lacking glucose, these lethal phenotypes can be rescued (20). We measured glycosome number and size in low-glucose medium (SDM79θ; 5 μM glucose) with the expectation that under these conditions, we could score phenotypes that would be lethal in cells grown in high glucose. In low-glucose medium, the increase in glycosome size upon Pex13.2 silencing was greater (Fig. 3B). Glycosomes had an average area of 0.22 μm^2^ in uninduced cells and 0.26 μm^2^ in induced cells. Similarly, the reduction of glycosome number after induction was more dramatic when cells were grown in low-glucose medium. Uninduced cells had an average of 23.5 glycosomes/100 nm^2^, while induced cells had 14.4 glycosomes/100 nm^2^. These results indicate that glycosome number and size were slightly but significantly affected (*P* values < 0.05) by Pex13.2 depletion, with induced cells having fewer but larger glycosomes.

We next used density gradients to determine the density of glycosomes from uninduced and induced parasites (Fig. 3C). In uninduced cells, the glycosome proteins aldolase and Pex14 were detected in fractions 10 to 17, while the ER protein BiP was enriched in fractions 1 to 10. In induced cells, the majority of BiP was still detected in fractions 1 to 10, while the glycosome proteins aldolase and Pex14 were detected in higher fractions (4 to 13) of lower density. These results indicate that the glycosomes of Pex13.2-deficient parasites are lighter than those in cells harboring wild-type levels of Pex13.2.

### Silencing Pex13.2 in PF parasites reduced import efficiency of PTS2 sequences.

**FIG 4:**
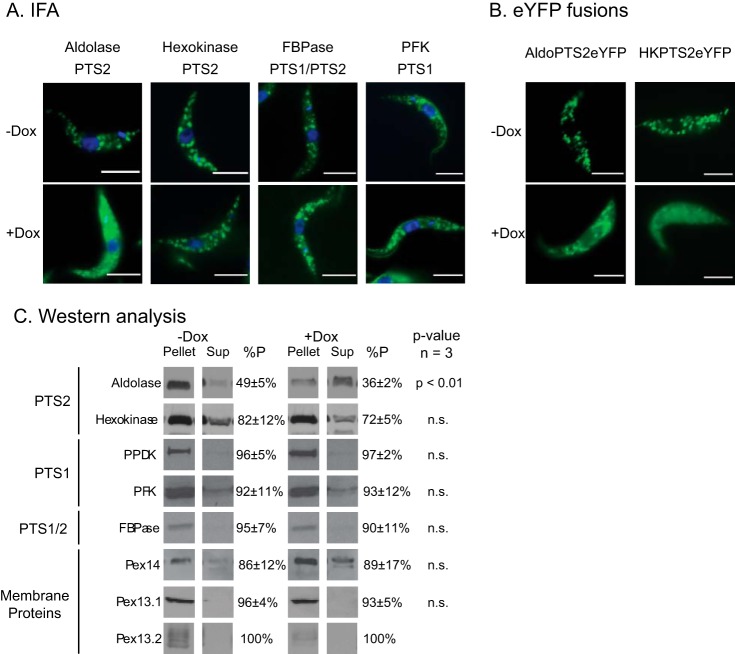
Silencing of Pex13.2 results in mislocalization of a subset of glycosome proteins. (A) IFAs of PF cells grown in the presence or absence of doxycycline (Dox). Cells were labeled with antibodies against aldolase, hexokinase, and fructose 1,6-bisphosphatase (FBPase) and detected using goat anti-rabbit secondary antibodies conjugated with Alexa Fluor 488 (green). Nuclear and kinetoplast DNA were stained with DAPI (blue). (B) Live-cell microscopy of cells constitutively expressing eYFP fused with the peroxisome targeting sequence 2 (PTS2) of aldolase or hexokinase (HK) (green). (C) Biochemical analysis of mislocalization. Cells were lysed by silicon carbide abrasive and were centrifuged to obtain an organelle-enriched pellet. Cytosol proteins in the supernatant were precipitated using 4 volumes of acetone, separated by SDS-PAGE, and analyzed by Western blotting. Mislocalization was quantified by densitometry of three biological replicates using ImageJ.

In higher eukaryotes, Pex13 functions in peroxisomal protein import, and silencing Pex13.2 in BF parasites compromises glycosome protein import (13). We used immunofluorescence assays (IFAs) to determine if Pex13.2 silencing altered glycosome protein import in PF parasites. In uninduced cells, the glycosome proteins aldolase, hexokinase (HK), fructose 1,6-bisphosphatase (FBPase), and phosphofructokinase (PFK) all localized to punctate structures characteristic of glycosomes (Fig. 4A). After 4 days of induction, IFA revealed an increase in the cytosolic staining of aldolase. In contrast, no change in staining of HK, FBPase, and PFK was observed.

To quantify the import efficiencies of each protein and identify mislocalization that is not detected by IFA, we used Western analysis. Lysates from uninduced and induced cells were centrifuged to obtain a membrane-rich fraction, and Western analysis and densitometry were used to calculate the percentage of each protein associated with membrane (glycosomes) and soluble (cytosolic) fractions (Fig. 4C). In uninduced cells, 49 ± 5% of aldolase was associated with the pellet compared to 36 ± 2% in induced cells. In contrast to that for aldolase, the percentages of FBPase, PFK, and HK localization associated with glycosome-enriched fractions did not change when Pex13.2 was silenced.

While both HK and aldolase have type 2 peroxisomal targeting sequences (PTS2s), we were surprised that only aldolase localization was affected by Pex13.2 silencing. To determine if the differences in HK and aldolase localization in response to Pex13.2 depletion was due to the particular PTS2 sequence of each protein, we followed the localization of a reporter protein, enhanced yellow fluorescent protein (eYFP), fused to either the PTS2 of aldolase (AldoPTS2eYFP) or the PTS2 of HK (HKPTS2eYFP). Fluorescence microscopy of living cells revealed that both AldoPTS2eYFP and HKPTS2eYFP were mislocalized to the cytoplasm in Pex13.2-deficient cells (Fig. 4B), indicating that both PTS2s from HK and aldolase were affected by Pex13.2 depletion. Import experiments shown here were performed with cells grown in minimal glucose. When grown in high glucose, the mislocalization of aldolase and HKPTS2eYFP were observed but to a lesser degree (data not shown).

### Pex13.2 partially colocalizes with Pex13.1 and is localized to discrete foci in glycosomes.

**FIG 5:**
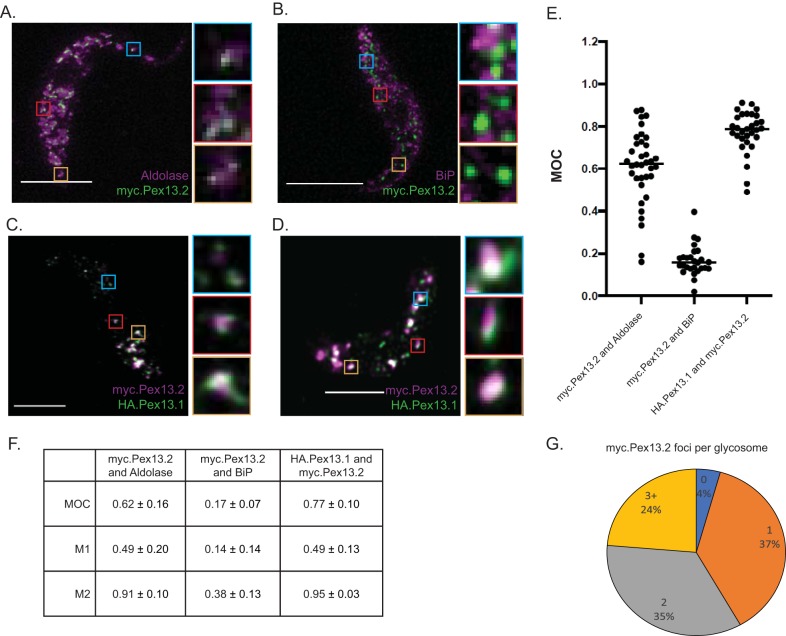
Superresolution imaging of myc.Pex13.2. (A) Representative images of cells expressing myc.Pex13.2 taken using a Leica SP8 confocal microscope equipped with HyD detectors. (A) Myc.Pex13.2 (green) and aldolase (magenta); (B) BiP (magenta) and myc.Pex13.2 (green); (C and D) myc.Pex13.2 (magenta) and HA.Pex13.1 (green). Bars, 5 μm. (E) Manders overlap coefficients for myc.Pex13.2-aldolase, myc.Pex13.2-BiP, and myc.Pex13.2-HA.Pex13.1 calculated from superresolution images using the FIJI distribution of Image J (36, 37). Each value represents a single cell image made up of 6 to 10 0.22-μm slices. (F) MOC, M1, and M2 values. Analysis was performed with 34 cells (myc.Pex13.2 and aldolase), 27 cells (myc.Pex13.2 and BiP), and 27 cells (HA.Pex13.1 and myc.Pex13.2). (G) Pie chart of the number of myc.Pex13.2 foci per glycosome.

IFAs, sucrose gradients, and membrane association experiments indicated that Pex13.2 is a glycosome membrane protein. Recent work in other systems has shown that components of the peroxisome import machinery localize to distinct regions of the peroxisome periphery (21). We used superresolution microscopy to determine if Pex13.2 exhibited similar localization. Antibodies against the matrix protein aldolase labeled punctate structures approximately 280 nm in diameter. In contrast, antibodies that recognize myc.Pex13.2 labeled smaller regions of the aldolase-positive structures, approximately 58 nm in diameter. We used Manders overlap coefficients (MOCs) to quantify the extent to which myc.Pex13.2 overlapped aldolase (Fig. 5E). MOC values range from 0 (no overlap) to 1 (complete overlap) (22, 23). The MOC for aldolase and myc.Pex13.2 was 0.62 ± 0.16, suggesting incomplete overlap. To better define the relationship between myc.Pex13.2 and aldolase staining, we calculated Manders M1 and M2 values, which reveal the extent to which pixels in one channel overlap the other. The M1 and M2 values were 0.49 ± 0.20 and 0.91 ± 0.10, respectively. These numbers indicate that 49% of the signal in channel 1 (aldolase) overlaps that in channel 2 (myc) and that 91% of the signal in channel 2 (myc) overlaps that in channel 1 (aldolase), and images reveal that myc.Pex13.2 is limited to a restricted portion of the aldolase-positive structures. Of the myc.Pex13.2-positive glycosomes, 37% had one focus, 35% had two foci, and 24% had 3 or more. Of aldolase-positive glycosomes, approximately 4% had no visible myc.Pex13.2 foci (Fig. 5G).

Coimmunoprecipitations indicated that HA.Pex13.1 and myc.Pex13.2 physically interact. Additionally, 2-dimensional gel electrophoresis suggested that these proteins form multiple, high-molecular-weight complexes. We next used superresolution microscopy to determine the extent to which these proteins overlap in cells (Fig. 4C and D). Image analysis revealed that the proteins exhibit some colocalization, with an MOC of 0.77 ± 10 (Fig. 4E) and M1 and M2 values of 0.49 ± 0.13 and 0.95 ± 0.03, respectively (Fig. 4F), indicating that 49% of Pex13.2 signal overlapped that of Pex13.1 while 95% of Pex13.1 overlapped Pex13.2. IFA revealed the presence of structures harboring HA.Pex13.1 but lacking detectable levels of myc.Pex13.2 in addition to Pex13.2 structures lacking detectable levels of HA.Pex13.1. These results indicate that the two proteins sometimes localize together but exhibit distinct localization.

## DISCUSSION

Kinetoplastids have unique peroxisome-like organelles called glycosomes that are essential to parasite viability. Like peroxisomes, glycosomes have highly conserved protein machinery that facilitates the import of proteins into the organelle from the cytoplasm where they are synthesized. During the import process, the cargo protein is bound by either Pex5 or Pex7, which bind to a peroxisome targeting sequence 1 (PTS1) or PTS2, respectively. The receptor-cargo complex then docks at the membrane through interactions with Pex13 and Pex14. While eukaryotes have a single Pex13, kinetoplastid parasites are unique in that they have two Pex13s, Pex13.1 and Pex13.2.

In previous work (13), Pex13.1 localized to glycosomes with the C-terminal SH3 domain on the cytoplasmic side of the organelle. The silencing of the protein resulted in glycosome protein import deficiencies, growth defects, and mislocalization of Pex14, and yeast two-hybrid studies suggested that Pex13.1 interacted with Pex14 and Pex5 (13). Pex13.2 was identified later, and silencing it in BF parasites resulted in glycosome protein mislocalization and cell death (14). In those studies, PF RNA interference cell lines could not be obtained. In two of three yeast two-hybrid screens, Pex13.2 interacted with Pex13.1. Interactions between Pex14 and Pex13.2 were not detected, although the authors indicated they could not rule out technical reasons for this lack of interaction.

While Pex13.1 and Pex13.2 are essential and nonredundant, it is unknown why kinetoplastids have two of these proteins when one is sufficient for most organisms. Because mislocalization of glycosome proteins is usually lethal, we hypothesize that Pex13.2 is necessary for the efficient import of proteins whose rapid sequestration in the glycosome is essential. Interference of glycosome biogenesis and protein import kills parasites (24, 25), and resolving the function of these novel Pex13s will likely reveal processes that can be exploited for pragmatic gain.

To understand the function of Pex13.2, we characterized the phenotype of Pex13.2-deficient cells, partially resolved the topology of Pex13.2, demonstrated that it binds both Pex13.1 and Pex14, and identified three import complexes. The model kinetoplastid, Trypanosoma brucei, alternates between the tsetse fly and a mammalian host. Previous work in mammalian bloodstream-form (BF) parasites revealed that silencing of Pex13.2 disrupts glycosome protein import (14). Here, we have characterized Pex13.2-deficient procyclic-form (PF) parasites that reside in the fly. This stage of the parasite is less sensitive to the disruption of glycosome function than BF, and we reasoned that we would observe phenotypes in this stage that are lethal in BF. Additionally, PF parasites are more tolerant of the expression of epitope-tagged glycosome proteins, which was required for these studies.

We found that Pex13.2 silencing resulted in mislocation of aldolase containing a PTS2, while the import of several PTS1 proteins was not affected. We were surprised to find that the localization of another PTS2 protein, HK, was not altered upon depletion of Pex13.2. There are two scenarios that may explain this observation. First, it may be that the two PTS2s differ in their relative strengths, with hexokinase PTS2 being a stronger targeting sequence that is less sensitive to Pex13.2 depletions than aldolase. There is precedence for such differences in PTS efficiencies. For example, replacement of the native low-efficiency PTS of Hansenula polymorpha catalase with a stronger high-efficiency PTS results in faster import of the protein from the cytoplasm. This increased import efficiency results in a decrease in overall catalase activity, as inactive aggregates are formed in the peroxisomes (26). It is hypothesized that the weaker native PTS allows more time for the protein to fold properly prior to import and is essential to maintaining high enzymatic activity. The finding that both HK PTS2 and aldolase PTS2 fused to the reporter eYFP exhibited similar mislocalization upon depletion of Pex13.2 suggests that the different behaviors of the two proteins is not a function of PTS2 efficiencies. We hypothesize that the lack of HK mislocalization in Pex13.2-deficient cells is the consequence of a cryptic PTS that targets HK to glycosomes when PTS2 targeting is disrupted. The presence of two independent PTSs has been demonstrated for the protein catalase A in Saccharomyces cerevisiae (27). Work is in progress to identify these cryptic targeting sequences.

Transmission electron microscopy revealed that Pex13.2-deficient cells had fewer but larger glycosomes, indicating that the protein is important for glycosome formation and/or proliferation as well as protein import. Glycosome morphology was not investigated in previous Pex13.2 studies (13, 18). The finding that the N-terminal YG-rich region of Pex13.2 is exposed to the cytoplasm is significant, as it is this portion of the protein that interacts with Pex7 during the docking process. It is reasonable to propose that silencing of Pex13.2 reduces the number of Pex7 docking sites, which leads to compromised import of PTS2 proteins. Our findings are consistent with previous studies in which Pex13.2 silencing in BF parasites resulted in protein import defects. We are pursuing *in vitro* binding assays to determine if recombinant Pex7 binds the YG-rich region of Pex13.2. However, our inability to obtain large amounts of truncated Pex13.2 has hindered these efforts.

We demonstrated that Pex13.2 interacts with Pex13.1 and Pex14, known members of the docking complexes, further supporting its designation as a member of the glycosome protein import machinery. The observation that the association of Pex13.1 and Pex14 with the glycosome membrane is not disrupted in Pex13.2-deficient parasites suggests it is not essential to the formation of the primary Pex13.1/Pex14 import complex and is the basis for our designating it an accessory protein.

While Pex13.2 was essential in BF parasites (13), we did not observe a growth defect in Pex13.2-deficient PF parasites. There are two possibilities for these different outcomes. First, it is possible that there is a relaxed requirement for Pex13.2 in PF parasites. Because BF parasites are more sensitive to glycosome disruption, this scenario is not unreasonable. Second, the differences in growth phenotype may be a consequence of incomplete Pex13.2 silencing, where the remaining Pex13.2 is sufficient to support growth. While possible, this is unlikely as the protein levels were reduced ∼96% upon induction.

There is limited information on the topology of Pex13.1 or Pex13.2. Previous work showed that the C terminus of Pex13.1 was on the cytoplasmic side of the glycosome (14). Our findings build a more complete picture of the import complex, revealing that the N termini of Pex13.1 and Pex13.2 are also on the cytoplasmic side of the glycosome. Unfortunately, multiple attempts to tag the C termini of Pex13.1 and Pex13.2 have been unsuccessful. While we cannot rule out a technical basis for this result, it may be that these regions are essential to critical aspects of Pex13 localization and/or function. The finding that Pex13.1, Pex13.2, and Pex14 interact in parasites validates and expands results obtained in yeast two-hybrid studies (13, 14) where Pex13.1 interacted with Pex14 and Pex5 and Pex13.2 interacted with Pex13.1 and Pex19. In these studies, we demonstrated that those interactions occur in parasites and that Pex13.2 interacted with Pex14, a combination that could not be tested in previous work due to technical issues.

In other eukaryotes, several high-molecular-weight peroxisome import complexes have been characterized (28). Previous to our work, the number, size, and composition of the docking complexes in any kinetoplastid were unknown. Here, we resolved three complexes. One very-high-molecular-weight complex contained Pex13.1 and Pex13.2 but lacked detectable levels of Pex14. Similar large Pex13 complexes of unknown function have been identified in yeast and mammalian cells (28). To our knowledge, complexes containing Pex14 but lacking Pex13 have not been reported in eukaryotes. At this point, it is unknown whether any or all of these complexes support glycosome protein import.

Glycosome density was reduced in Pex13.2-deficient cells. This has been observed before in peroxisome mutants (29) and may be a function of reduced protein import or the result of having reduced amounts of Pex13.2 in the glycosome membrane. Mass spectrometry is necessary to discriminate between the two possibilities and determine if the import of other proteins is altered in Pex13.2-deficient cells.

We found that Pex13.2 localized to discrete regions of the glycosome periphery. Such asymmetric distribution of peroxins has been reported in other systems (21). Studies are ongoing to determine if other components of the import machinery exhibit similar suborganelle localization. This Pex13.2 localization pattern along with the reduction of glycosome number and the increase in size suggest that Pex13.2 may be involved in glycosome division. The ability to resolve individual organelles via superresolution microscopy is a critical advance that will allow us to identify residues required for this unique localization and assess the biological significance of this localization. Attempts to demonstrate this localization via immunoelectron microscopy has been unsuccessful, because antibodies that recognize these proteins do not work under fixation conditions required for this technology.

Here, we have identified three glycosome import complexes and demonstrated that Pex13.2 is a bona fide member of the docking complex in T. brucei and necessary for efficient import of PTS2 proteins. Current work is focused on resolving the composition and function of each complex. Because disruption of the protein interactions that facilitate glycosome protein import is toxic to T. brucei, a detailed understanding of composition and function of complexes that facilitate these processes is crucial to our ability to target them for therapeutic purposes.

## MATERIALS AND METHODS

### Cell culture and transfection of T. brucei.

Procyclic-form (PF) 2913 and bloodstream-form (BF) 9013 expressing T7 polymerase and tetracycline (tet) repressor (30) were maintained in SDM79 (or the minimal glucose medium SDM79θ containing 5 μM glucose) (31) and HMI-9, respectively. Expression vectors for epitope-tagged proteins were generated by cloning the open reading frame of Pex13.2 into the pXS2 (PCF) vector or pXS6 (BSF) vectors possessing either a blasticidin resistance or puromycin resistance gene (32). For transfection, 20 μg plasmid DNA was linearized (pXS2, pXS6: MluI; pZJM: NotI) and electroporated in 4-mm cuvettes (Bio-Rad GenePulser Xcell; exponential, 1.5 kV, 25 μF). Twenty-four hours after electroporation, culture medium was supplemented with the appropriate drug for selection: 15 μg/ml G418, 50 μg/ml hygromycin, 2.5 μg/ml phleomycin, 1 μg/ml puromycin, or 10 μg/ml blasticidin. RNA interference (RNAi) cell lines were generated by cloning nucleotides 41 to 441 of *Tb*Pex13.2 into the inducible pZJM vector possessing dual opposing T7 promoters and a phleomycin resistance marker (33). Usually, we grow RNAi cell lines in tet-free medium to reduce leaky expression from RNAi plasmids. However, this was not required for these cell lines, as we did not observe leaky expression.

### Pex13.2 antibody production.

Polyclonal guinea pig antiserum was generated against truncated recombinant Pex13.2 (Thermo Scientific). Amino acids 2 to 150 of Pex13.2 fused to an N-terminal His_6_ tag were expressed using the pQE30 expression system (Qiagen) and purified using a nickel-nitrilotriacetic acid (Ni-NTA) column under denaturing conditions using 8 M urea as described (34) for use as antigen.

### Growth curves.

Cells possessing the tetracycline inducible pZJM:Pex13.2 vector were seeded at 10^5^ cells/ml in SDM79 (PF) and induced with 1 μg/ml doxycycline. Cells were allowed to grow to a density of 5 × 10^6^ cells/ml prior to passing back to 1 × 10^5^. Culture density was monitored by flow cytometry at 24-h intervals using an Accuri C6 flow cytometer (BD Biosciences).

### Sucrose gradient fractionation and Western blot.

Large-scale sucrose gradient fractionation (Fig. 1) was carried out as described previously (35). Small-scale fractionations (Fig. 3) were separated as described previously (35) with the following modifications: the postnuclear lysate was separated on a 13-ml 20% to 40% linear OptiPrep gradient at 30,000 RPM for 17 h in a Beckman SW-40Ti rotor at 4°C (acceleration, 9; deceleration, coast); 500-μl fractions were taken from the top of the gradient, and the protein concentration was determined by bicinchoninic acid (BCA) assay (Thermo Fisher). Protein from each fraction (2.5 μg) was separated by SDS-PAGE and analyzed by Western blotting with antibodies against the glycosomal proteins: aldolase (1:20,000), Pex13.1 (1:10,000), Pex13.2 (1:10,000), Pex11 (1:4,000) provided by Christine Clayton (Zentrum für Molekulare Biologie der Universität Heidelberg, Germany) (16), PFK (1:10,000) and FBPase (1:10,000) provided by Paul Michels (University of Edinburgh, UK), and the ER protein BiP (1:100,000) provided by Jay Bangs (University at Buffalo, Buffalo, NY) (32).

### Protease protection assays.

Protease protection assays were carried out using a modified protocol previously described (16). Cells (10^6^) were harvested at 800 × *g* for 10 min, washed once in PBS (150 mM NaCl, 1 mM KH_2_PO_4_, 5.6 mM Na_2_HPO_4_, pH 7.4), once in STE buffer (250 mM sucrose, 25 mM Tris-HCl [pH 7.4], 1 mM EDTA), and resuspended in 98 μl ice-cold STEN buffer (STE buffer supplemented with 150 mM NaCl) and 0.1 mM phenylmethylsulfonyl fluoride (PMSF). Cells were permeabilized with 2 μl 1 mg/ml digitonin (final concentration of 0.02 mg/ml), vortexed for 5 s, and incubated at room temperature for 4 min. Following permeabilization, cells were centrifuged at 20,000 × *g* for 2 min and resuspended in 85 μl STEN buffer. Pellets were treated with either 10 μl water or Triton X-100 (1% [vol/vol] final) and either 5 μl water or 2 mg/ml proteinase K. Reaction mixtures were incubated on ice for 30 min and stopped by addition of 10% (wt/vol) trichloroacetic acid (TCA). Precipitated proteins were centrifuged at 17,000 × *g* for 10 min and washed once with acetone before being resuspended in cracking buffer (CB; 10% glycerol, 2% SDS, 2% β-mercaptoethanol, 100 mM Tris [pH 6.8], 0.1% bromophenol blue) and boiled at 100°C. Proteins were then analyzed by SDS-PAGE and Western blotting.

### Membrane association assays.

Membrane association assays were carried out as previously described (16). For extraction of membrane proteins, 10^7^ cells were centrifuged at 800 × *g* for 10 min and resuspended in 300 μl of ice-cold low-salt buffer for 15 min (5 mM Tris-HCl [pH 7.8], 1 mM EDTA, 0.1 mM PMSF, 4 μg/ml leupeptin). Cells were then passed through a pipette tip 10× and centrifuged at 20,000 × *g* for 30 min at 4°C. The insoluble pellet was resuspended in 300 μl high-salt buffer (25 mM Tris-HCl [pH 7.8], 0.5 M KCl, 1 mM EDTA, 0.1 mM PMSF, 4 μg/ml leupeptin) and incubated on ice for 15 min. After incubation, samples were centrifuged again at 20,000 × *g* for 30 min at 4°C. The insoluble pellet was resuspended in 300 μl 0.1 M Na_2_CO_3_ and incubated for 30 min on ice. Samples were then centrifuged at 120,000 × *g* for 1 h at 4°C with a 500-μl cushion of 0.1 M Na_2_CO_3_, 0.25 M sucrose in a Beckman TLA100.3 rotor. Supernatant protein was precipitated by 10% (wt/vol) TCA and washed once with acetone before being resuspended in CB. Samples were then separated by SDS-PAGE and analyzed by Western blot.

### Live-cell microscopy.

Cells expressing either AldoPTS2eYFP or HKPTS2eYFP were washed once with PBS, mounted on a slide, and visualized using a Zeiss Axiovert 200M inverted fluorescence microscope with a 100× lens objective (numerical aperture [NA] 1.3). Images were analyzed using AxioVision software version 4.8.2.

### Immunofluorescence microscopy.

All steps were performed at room temperature. Cells were harvested (800 × *g*, 10 min), washed once with PBS, fixed with 2% paraformaldehyde in PBS for 30 min, and allowed to settle on slides for 30 min. Adhered cells were washed once with wash solution (0.1% normal goat serum in PBS) and permeabilized with 0.5% Triton X-100 for 30 min. Following permeabilization, cells were washed twice with wash solution and blocked with 10% normal goat serum (NGS) in PBS with 0.1% Triton X-100. Primary antibodies were diluted in block solution (aldolase, 1:500; HK, 1:500; FBPase, 1:1,000; PFK, 1:500; mouse anti-HA [BioLegend], 1:50; myc-tag [71D10, Cell signaling], 1:100; C-myc [Thermo Fisher, 9E10], 1:500) and incubated with cells for 1 h. Following the incubation with primary antibody, slides were washed 3× with wash solution and incubated 1 h with secondary antibody (goat anti-mouse Alexa Fluor 488, 1:1,000; goat anti-rabbit Alexa Fluor 568 or Alexa fluor 647, 1:1,000; Thermo Fisher) in block solution. Wide-field images were taken using a Zeiss Axiovert 200M with a 100× lens objective (NA, 1.3), and analyzed using AxioVision software version 4.8.2. Samples used in superresolution microscopy were stained with the primary antibodies as indicated above and secondary antibodies (goat anti-mouse Alexa Fluor 488 and 647 at a 1:1,000 dilution) and images were obtained using a Leica SP8X microscope equipped with a HyD detector and 63× lens objective (NA, 1.4). Manders overlap coefficients (MOC; M1 and M2) were calculated using the ImageJ FIJI (version 1.52) Manders coefficients plug in (22, 36, 37).

### Electron microscopy processing and imaging.

Cells were harvested (5 × 10^7^, 800 × *g*, 10 min), washed three times with PBS, and fixed (2% paraformaldehyde, 2.5% glutaraldehyde in 100 mM phosphate buffer [pH 7.4]). Cells were stored at 4°C for no longer than 2 days before being processed as described previously (2). Glycosome area measurements were performed using FIJI. The area of visible glycosomes for 15 fields was measured using the measure tool. To calculate the glycosome area as a percentage of cell area, the area of glycosomes from each cell was summed and divided by the total cell area visible.

### Biochemical analysis of glycosome protein localization.

Cells were seeded at 1 × 10^5^ cells/ml, and RNAi against Pex13.2 was induced with 1 μg/ml doxycycline. After 4 days of induction, cells were harvested (800 × *g*, 10 min) and washed once with PBS. Cells (2 × 10^7^) were lysed using 1 volume wet weight silicon carbide abrasive, and breakage was confirmed by microscopy. The abrasive was removed by centrifugation (100 × *g*, 1 min), and the supernatant was transferred to a new tube, followed by removal of nuclei (1,000 × *g*, 15 min). The supernatant was transferred to a new tube, and the organelle-rich fraction was separated from the cytosolic fraction by centrifugation (17,000 × *g*, 15 min). Cytosolic proteins were precipitated with 4 volumes of acetone, incubated on ice for 1 h, and centrifuged at 17,000 × *g* for 10 min at 4°C. The pelleted organelle-rich fraction and cytosolic proteins were separated by SDS-PAGE, and proteins were detected by Western blotting.

### Coimmunoprecipitations.

Cells (2 × 10^9^) constitutively expressing HA.Pex13.1 and Myc.Pex13.2 were harvested at 800 × *g* for 10 minutes and washed two times with PBS. Cells were incubated with 2.5 M DTSSP (3,3’-dithiobis[sulfosuccinimidylpropinoate]) for 30 min, Tris pH 7.5 was added to a final concentration of 50 mM, and cells were lysed by resuspending in hypotonic lysis buffer 1 (HB1) (100 μM TLCK [tosyl phenylalanyl chloromethyl ketone], 1 μg/ml leupeptin) and incubating on ice for 20 min. An equal volume of hypotonic lysis buffer 2 (HB2) was added (100 mM Hepes-KOH pH 8, 50 mM KCl, 10 mM MgCl_2_, 20% [vol/vol] glycerol, 100 μM TLCK, 1 μg/ml leupeptin). Lysates were passed through a 28-g needle five times and centrifuged (17,000 × *g*, 10 min, 4°C), and clarified lysate (L1) was transferred to a fresh tube. The pellet was resuspended in equal volumes HB1 and HB2, supplemented with 0.5% IGEPAL, incubated on ice for 20 min, and centrifuged (17,000 × *g*, 10 min, 4°C), and clarified lysate (L2) was transferred to a fresh tube. Each lysate (L1 and L2) was aliquoted into two tubes and 100 μl magnetic anti-HA or anti-Myc beads was added. Samples were incubated rotating overnight at 4°C, beads were separated using a magnetic rack, and samples were washed three times with 5× TBS-T (100 mM Tris pH 7.5, 750 mM NaCl, 0.5% Tween 20), washed 1× in nanopure water, and resuspended in 100 μl nanopure water. Samples were then analyzed by SDS-PAGE and Western blotting.

### 2-Dimensional blue native/SDS-PAGE.

An organelle-enriched pellet from 10^8^ cells was prepared as previously described and resuspended in 1× sample buffer (1% n-dodecyl-β-d-maltoside, 0.75 M 6-aminocaprotic acid, 0.5% Coomassie brilliant blue-G250, 50 mM Bis-Tris pH 7.0, 0.5 mM EDTA). Samples were then incubated rotating at 4°C for 1 h and centrifuged (100,000 × *g*, 15 min, 4°C) to remove insoluble material. The supernatant was resolved by blue-native PAGE (BN; Invitrogen Novex NativePage 4–16% Bis-Tris gel; blue cathode buffer: 50 mM Tricine-NaOH pH 7.0, 15 mM Bis-Tris-HCl, 0.02% Coomassie brilliant blue G-250; anode buffer: 50 mM Bis-Tris-HCl pH 7.0). When dye front migrated ~1/3 through the gel, cathode buffer was exchanged for a clear cathode buffer (without CBB). To analyze in second-dimension SDS-PAGE, individual lanes were excised, incubated in warm SDS-PAGE running buffer, and overlayed on a 12% SDS-PAGE gel. After transfer to nitrocellulose membranes, proteins were analyzed by Western blotting using antibodies against Pex13.1 (1:5,000), Pex13.2 (1:10,000), and Pex14 (provided by Armando Jardim, McGill University, Quebec, Canada; 1:20,000).
